# Effects of Specific Bioactive Collagen Peptides in Combination with Concurrent Training on Running Performance and Indicators of Endurance Capacity in Men: A Randomized Controlled Trial

**DOI:** 10.1186/s40798-023-00654-9

**Published:** 2023-11-07

**Authors:** Simon Jerger, Patrick Jendricke, Christoph Centner, Kevin Bischof, Jan Kohl, Simon Keller, Albert Gollhofer, Daniel König

**Affiliations:** 1 Department of Sport and Sport Science, University of Freiburg, Schwarzwaldstraße 175, 79117 Freiburg, Germany; 2 Praxisklinik Rennbahn, Muttenz, Switzerland; 3 Department for Nutrition, Exercise and Health, Centre of Sports Science, University of Vienna, Auf Der Schmelz 6, 1150 Vienna, Austria; 4 Department for Nutrition, Exercise and Health, Faculty of Life Sciences, University of Vienna, Josef-Holaubek-Platz 2, 1090 Vienna, Austria

**Keywords:** Running endurance performance and capacity, Lactate threshold, Body composition, Endurance training, Collagen peptides, Protein supplementation

## Abstract

**Background:**

First evidence indicates that the supplementation of specific collagen peptides (SCP) is associated with a significant improvement in running performance in physically active women; however, it is unclear if the same is true in males. The purpose of the present study was to investigate the effects of a concurrent training program including 60 min of continuous moderate intensity running training and 15 min of dynamic resistance training combined with supplementation of SCP on parameters of running performance in moderately trained males.

**Methods:**

In a double-blind, placebo-controlled, randomized trial, participants performed a 12 weeks concurrent training and ingested 15 g of SCP [treatment group (TG)] or placebo [control group (CG)] daily. Before and after the intervention, running endurance performance was measured by a 1-h time trial on a running track. Velocity at the lactate threshold (*V*_LT_) and at the individual anaerobic threshold (*V*_IAT_) were assessed on a treadmill ergometer. Body composition was evaluated by bioelectrical impedance analysis.

**Results:**

Thirty-two men (28.4 ± 5.2 years) completed the study and were included in the analysis. After 12 weeks, TG had a statistically significant (*p* ≤ 0.05) higher increase in running distance (1727 ± 705 m) compared to the CG (1018 ± 976 m) in the time trial. *V*_LT_ increased in the TG by 0.680 ± 1.27 km h^−1^ and slightly decreased by − 0.135 ± 0.978 km h^−1^ in the CG, resulting in statistically significant group differences (*p* ≤ 0.05). A significantly higher improvement in *V*_IAT_ (*p* ≤ 0.05) was shown in the TG compared with the CG only (1.660 ± 1.022 km h^−1^ vs 0.606 ± 0.974 km h^−1^; *p* ≤ 0.01). Fat mass decreased (TG − 1.7 ± 1.6 kg; CG − 1.2 ± 2.0 kg) and fat free mass increased (TG 0.2 ± 1.2 kg; CG 0.5 ± 1.3 kg) in both groups with no significant group differences.

**Conclusion:**

In summary, supplementation with 15 g of SCP improved running performance in a 1-h time trial and enhanced indicators of endurance capacity at submaximal exercise intensities such as an increased velocity at the lactate as well as the anaerobic threshold more effectively than CT alone.

*Trial registration*: ETK: 123/17; DRKS-ID: DRKS00015529 (Registered 07 November 2018—Retrospectively registered); https://drks.de/search/de/trial/DRKS00015529

## Background

Resistance training and aerobic training promote distinct positive adaptations of the muscular and cardiovascular system [1]. While resistance training best promotes myofibrillar protein synthesis [2], aerobic training has a positive impact on favorable adaptations within the cardiovascular system and endurance performance. The increase in aerobic capacity is accomplished by augmentations in vascular and mitochondrial density or upregulation of enzymes of aerobic metabolic pathways, for instance [3]. Appropriate improvement of both musculoskeletal and cardiovascular performance is crucial not only for athletes [4], but also for the prevention or treatment of age-related chronic diseases as sarcopenia, diabetes or cardiovascular diseases [5, 6]. Therefore, concurrent training (CT)—a combination of resistance and endurance training—is recommended to improve muscular performance and cardiovascular health and to reduce the risk of cardiometabolic diseases [7].

However, CT often results in lower metabolic, strength and muscle hypertrophy adaptations than endurance or resistance training alone [8]. Evidence indicates that this is partly mediated via the phenomenon of interference and it has been speculated that protein supplementation could mitigate the interference effect of endurance training on muscle hypertrophy [9–11].

Both training concepts initiate signaling pathways which could trigger adaptations on either muscular or mitochondrial protein synthesis [12]. This fact highlights the potential benefit of dietary proteins on CT. In this context, the acute and chronic effects of protein supplementation and CT on skeletal muscle adaptations, anabolic signaling pathways and aerobic performance have been examined previously [7]. Based on the results of this systematic review, the authors concluded that protein ingestion acutely increases myofibrillar, but not mitochondrial, protein synthesis rates during post-exercise recovery. In addition, they found that protein supplementation could further enhance training-mediated increases in skeletal muscle mass and strength/power, but not whole-body aerobic capacity following longer-term concurrent training.

It can be assumed that the effects of CT on hypertrophy, strength and endurance capacity depend on modality, sequence, frequency, and duration of the endurance and resistance component [8]. Therefore, the efficacy of protein supplementation to improve the adaptive metabolic and structural response to the endurance or resistance training component also depends on the composition of the CT.

Furthermore, the type of supplement might influence the adaptions. In contrast to protein supplements rich in the essential amino acid leucine (e.g., whey, casein), SCP shows a distinctive amino acid profile with lower levels of essential amino acids [13], but high concentrations of glycine, hydroxyproline and proline comparable to the presence in collagen, the most abundant protein within the musculoskeletal system and principle component of the extracellular matrix (ECM) [14]. Recently, positive effects of supplementation with specific collagen peptides (SCP) in combination with long-term resistance training programs on body composition, muscle mass and muscle strength have been reported in various populations, including men and women of different age groups and training status (untrained or recreationally active) [13, 15–18]. In addition, a beneficial effect on bone mineral density has been shown postmenopausal women with a reduced bone mineral density [19]. The effects of SCP in combination with different CT regimes have been less investigated so far.

In a recent meta-analysis, only one study has particularly addressed the effect of SCP supplementation in combination with concurrent training. In this study, supplementation with SCP in combination with CT focused on endurance training resulted in a significant increase in running endurance performance and fat free mass in recreationally active women compared to a control group [20].

Furthermore, supplementation with SCP has been shown to increase fat free mass but also to reduce fat mass following resistance exercise [13, 15, 17]. Most recently, resistance training together with SCP revealed a significantly higher upregulation of key anabolic pathways in human skeletal muscle 4 h following an acute resistance training compared to the same training and placebo [21]. Therefore, there is increasing evidence that SCP could improve important functional and structural properties within skeletal muscles.

There are presently little data in young and healthy men and evidence indicates that men and women could respond differently to the stimulation of muscle-specific signaling proteins [22]. Investigating the effects of SCP supplementation on adaptations by CT could be of importance to both athletes and public health, as recent research presented above suggests positive effects on endurance performance as well as health parameters such as fat mass. The aim of this study was, therefore, to investigate the effects of SCP and CT on running performance, aerobic and anaerobic threshold in an incremental treadmill ergometry and body composition in recreationally active men. With respect to the results of a previous study [20], we hypothesized that the combination of SCP ingestion and CT will improve indicators of endurance capacity and body composition in men as well.

Therefore, the primary endpoint of the study was the impact of post-exercise protein supplementation with 15 g per day of SCP vs. placebo on time trial performance. Furthermore, metabolic parameters in the incremental running test and, in addition, body composition were compared between groups as secondary endpoints.

## Methods

### Study Design

The study was designed as a monocentric, prospective, placebo-controlled, double-blinded trial conducted at the University of Freiburg, Germany. Approval of the study was obtained from the local ethical committee of the University of Freiburg (ETK: 123/17), and all procedures were in accordance with the guidelines set by the Declaration of Helsinki of 1975 as revised in 1983. The trial was registered at the German Clinical Trials Register (DRKS-ID: DRKS00015529).

Following written informed consent, participants completed a screening to ensure that the inclusion criteria were met and that there were no risk factors that might be aggravated by the intervention program. Using a web-based random number generator, participants were randomly assigned to the group receiving 15 g SCP or 15 g placebo on a daily basis for 12 weeks. In addition, participants of both groups performed a 12-week CT three times a week. Table 1 illustrates the time schedule of the study.

**Table 1:** Schematic overview of the study design

Steps	Week															
	− 2	− 1	0	1	2	3	4	5	6	7	8	9	10	11	12	13
Screening and Randomization	x															
Time Trial (FAM)		x														
Incremental Running Test (FAM)		x														
Bioelectrical Impedance Analysis	x		x													x
Time Trial			x													x
Incremental Running Test			x													x
Physical Activity	x															x
Dietary Intake	x															x
Training & Supplementation				x	x	x	x	x	x	x	x	x	x	x	x	

*FAM* Familiarization, *Crosses* Measurement points or weeks with 3 training sessions and daily supplementation of SCP/PLA

### Participants

A total of *n* = 50 men aged between 18 and 40 years with a BMI between 18 and 26 kg m^−2^ and a body fat percentage of > 10% were recruited. Sample size was based on a previous study including recreationally active women who underwent the same intervention—12 weeks of CT and supplementation with SCP [20]. A post hoc power calculation (G*Power 3.1.9.2) for the main parameter, time trial performance, confirmed that a sufficient number of participants were included, as it resulted in an actual power value of 0.996. In order to avoid overuse injuries and high failure rates and to ensure that participants are receptive for exercise-induced adaptations, moderately endurance-trained male runners with an experience of 1–2 training sessions of 1 h each per week were included in the study.

Furthermore, participants were only eligible if they were free of acute and chronic diseases such as cardiovascular, metabolic or renal diseases in accordance with the recommendation of American College of Sports Medicine [23]. Participation was not possible if collagen peptides or similar nutritional supplements have been taken in the previous 6 months. Besides a comprehensive anamnesis and physical examination, blood was analyzed for safety variables. Blood parameters (e.g., creatinine, urea nitrogen, creatine-kinase, erythrocyte sedimentation rate) were chosen to screen the organic and muscular health status of the subjects.

At a preliminary screening, the inclusion and exclusion criteria were checked using the anthropometric and questionnaire data, results from blood testing as well as the medical examination.

### Time Trial

The primary endpoint of the current investigation was to compare changes in running distance during a time trial performance on a 400m outdoor running track between study groups. For that purpose, participants underwent a 1-h time trial by covering the longest distance possible on a 400 m track to determine the running endurance performance. Runners were not aware of their performance times, heart rate (HR), or covered distance, but they were given a verbal reminder every 10 min and asked for their rating of perceived exertion (RPE) [24–26] on a 6–20 Borg Scale [27] to ensure comparable inter group exertion levels. The covered distance and HR were continuously tracked by a global positioning system (Polar M200, Kempele, Finland). The reliability and accuracy of GPS units from this manufacturer have been confirmed [28], and running distance was assessed with the exact same model in previous experiments [20]. All measurements were conducted in the morning with the objective of maintaining similar experimental conditions throughout. Temperatures ranged from 17 to 25 °C and humidity were between 50 and 60%.

### Incremental Running Test and Blood Lactate Analysis

In addition to the time trial, the incremental running test and blood lactate analysis were performed to draw conclusions on the endurance capacity between the study groups [26]. Both measurements were performed using a procedure, which has previously been described in detail [20]. During the incremental test on the treadmill (hp cosmos quasar®, Nussdorf-Traunstein, Germany), participants were instructed to give their RPE. Moreover, HR was monitored throughout all tests using a HR monitor (Polar M 200, Finland). Starting with 6 km h^−1^ the velocity was increased by 2 km h^−1^ every 3 min until exhaustion. Capillary blood samples were collected from the hyperemized earlobe [29] at rest, every 3 min and at exhaustion and analyzed using Biosen Glucose and Lactate analyzer (EKF diagnostics GmbH, Barleben/Magdeburg, Germany). As the first measurable increase in blood lactate concentration during the incremental running test, the lactate threshold (LT) was automatically evaluated by the computer software (Ergonizer 4.7.4, Freiburg, Germany). The individual anaerobic threshold (IAT) was determined as the velocity at a net increase in lactate concentration 1.5 mmol l^−1^ above the lactate concentration at LT [30]. In addition, HR and RPE were continuously recorded. Incremental running tests were conducted in an air-conditioned laboratory with temperature set at 20°C and relative humidity of 50%.

### Bioelectrical Impedance Analysis

Fat free mass (FFM) and fat mass (FM) as well as body weight were measured after a 12-h overnight fast using bioelectric impedance analysis (BIA). According to the recommendations of the European Society for Clinical Nutrition and Metabolism, all measurements were collected in a standardized way in order to reduce biological and technical error [31, 32]. Participants were instructed to avoid exercising (48 h), consuming alcohol (48 h) and caffeine (12 h). Participants’ body composition was measured on the BIA scale (seca© 274, Hamburg, Germany) which involved entry of the participant’s age, height to the nearest 1 mm to calculate the body mass index (BMI), and male gender. Still wearing the skin-tight clothing, participants stood on the scale barefoot and grasped the handle electrodes for ∼10 s until the process was completed. Metal and accessories were removed. In addition, each individual was asked to void their bladder prior to testing. The BIA system has shown to have acceptable within-session reliability (coefficient of variation < 2%) [33].

### Test Meal

In the laboratory 2 h before the exercise tests, a standardized meal was consumed [34] after a 12 h overnight fasted state. In order to achieve the best results in trials, test food contained 1 g per kg body weight of carbohydrate and was similar for all participants in terms of macronutrient content (percentage of energy).. It consisted of wholegrain flakes and semi-skimmed milk (1.5% fat). The individual amount of consumed flakes and milk was chosen to meet the macronutrient requirements according to body weight and complemented by water to 650 ml in total [35].

### Test Product Supplementation

The intervention beverages consisted of either 15 g SCP (PeptENDURE®, Gelita AG, Eberbach, Germany, for amino acid composition see Table 2) or 15 g placebo (silicon dioxide), with one half ingested 2 h before and the other half immediately after each training session in 250 ml of water. This should allow optimal use of the time window with the highest bioavailability of the supplement during the exercises and the recovery phase [36]. On days without training, participants were instructed to consume the test product supplementation at the same time as in the day before. Both test products did not differ in color, flavor and solubility. The supplement used is recognized by the US Food and Drug Administration as General Recognized and Safe.

**Table 2:** Amino acid composition PeptENDURE®

Amino acid	Weight (%)
Alanine	8.6
Arginine	7.3
Aspartic acid	5.8
Glutamic acid	10.2
Glycine	22.2
Histidine	1
Isoleucine	1.4
Leucine	2.7
Lysine	3.6
Hydroxylysine	1.6
Methionine	0.9
Phenylalanine	2.1
Proline	12.7
Hydroxyproline	11.9
Serine	3.2
Threonine	1.8
Tyrosine	0.8
Valine	2.4

All of the researchers and participants were blinded to which administration was consumed during the study.

### Physical Activity and Dietary Intake

The Freiburg Questionnaire of Physical Activity was used to report frequencies and time of additional physical and sports activity before and during the intervention [37]. The CT program was not included in the activity record. Based on the information provided in the questionnaire, the activity-related energy expenditure was determined.

In addition, all participants were asked to complete a 3-day dietary record, which included two weekdays and 1 day at the weekend, both before and after the intervention. The participants were instructed to record their total nutrition intake exclusive of the supplements. The dietary records were analyzed for daily energy and macronutrient intake using Nutriguide 4.6 (Nutri-Science GmbH, Freiburg, Germany).

### Concurrent Training Protocol

For the duration of the intervention, participants performed a combined endurance and resistance CT on three non-consecutive days of training each week. Each training session was supervised by experienced exercise instructors at the University of Freiburg.

The participants performed the 60-min endurance training on a 400 m track, which included 60 min of continuous moderate intensity running training on the basis of the World Health Organization (WHO) global physical activity guidelines [38]. In order to improve running endurance performance and capacity, subject’s intensities were elevated from 80% *V*_IAT_ in weeks 1–4 to 85% *V*_IAT_ in weeks 5–8 and 90% *V*_IAT_ in weeks 9–12 [39, 40].

Distance covered, heart rate, and RPE were recorded in all endurance training sessions by the experienced exercise instructors that supervised the training sessions. The endurance training was preceded by a 15 min resistance training program. In compliance with the training protocol of Klika and Jordan, the dynamic resistance training consisted of 3 sets of squats, lunges and one legged heel rises using the subject’s bodyweight [41]. Throughout the study, the number of repetitions gradually increased from 20 repetitions in weeks 1–4 to 25 repetitions in weeks 5–8 and finally to 30 repetitions in weeks 9–12. Exercises were trained with a total execution speed of 2 s in equal parts in the concentric and eccentric phases. A 30-s rest period was given between sets to ensure adequate recovery [42, 43].

### Statistical Analysis

Only per protocol analyses were included in the evaluation process using IBM SPSS Statistics (IBM SPSS Statistics for Windows, Version 25.0. Armonk, NY: IBM Corp.). All data are expressed as mean (M) ± standard deviation (SD) in tables and figures. All the tests were performed as two-sided tests, and the significance level was set at α = 0.05. Since data showed a normal distribution according to the results of a Kolmogorov–Smirnov test, parametric statistics were used. The homogeneity of the baseline values between study groups was checked via unpaired *t*-tests. Different developments between the groups over time were compared by using a mixed analysis of variance (ANOVA) of absolute pre- and post-values. The factors were group [treatment group (TG) and control group (CG)] and time (pre- and post-intervention levels). The significance of changes from baseline to post-intervention in the respective endpoints within groups was analyzed with the paired sample *t*-test. As a magnitude of the change in the respective outcomes, the effect size partial eta-square (*η*_p_^2^) was calculated (small effect: *η*_p_^2^ > 0.01, medium effect: *η*_p_^2^ > 0.06, large effect: *η*_p_^2^ > 0.14).

## Results

### Subject Characteristics

**Fig. 1:**
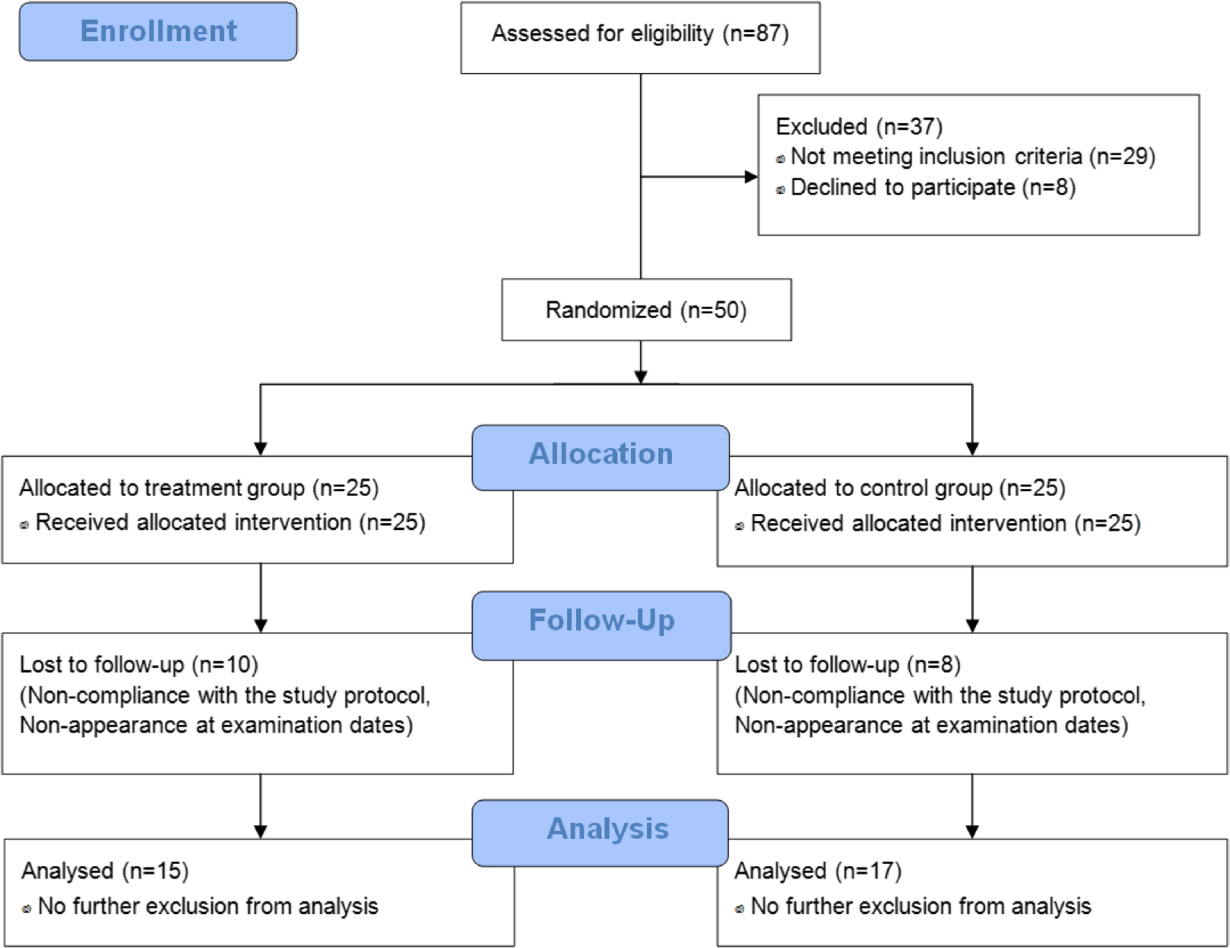
CONSORT flow diagram

Eighteen participants dropped out during the study because they missed too many training sessions or were unable to perform their training regimen adequately due to illness, injury or other reasons. Dropouts resulting from side effects of the supplemented SCP or the placebo did not occur. Furthermore, no pathological findings were observed in the routine blood test at baseline and following the interventions. As shown in Fig. 1, 32 participants (TG: *n* = 15 vs. CG: *n* = 17) completed the investigation and were included in the per protocol analysis.

Baseline anthropometric characteristics of both groups are summarized in Table 3. No statistically significant differences were identified between TG and CG at baseline for any of the assessed variables.

**Table 3:** Baseline anthropometric characteristics

Variables	TG (*n* = 15)	CG (*n* = 17)
Age (years)	28.6 ± 5.0	28.3 ± 5.6
Body height (cm)	180.5 ± 6.6	180.8 ± 6.9
Body mass (kg)	78.5 ± 9.2	75.6 ± 7.7
BMI (kg m^−2^)	24.0 ± 1.8	23.1 ± 1.6

*TG* Treatment group, *CG* Control group, *BMI* Body mass index, *p* value Independent *t*-tests, no significant between-group differences were found

### Efficacy Endpoints

The primary and secondary outcomes of the study participants are summarized in Table 4. No significant baseline differences between the study groups were detected in any outcome of the study.

**Table 4:** Changes in time trial performance, lactate thresholds, body composition, before (Pre) and following (Post) supplementation with SCP (TG) or placebo (CG) in combination with CT

Variables	TG (*n* = 15)		CG (*n* = 17)		*p* value (mixed ANOVA)		
	PRE	POST	PRE	POST	*T*	*G*	*T* × *G*
Time trial (m)	10,963 ± 1254	12,690 ± 1223***	10,735 ± 1448	11,753 ± 1313***	≤ 0.001	n.s	≤ 0.05
V_LT_ (km h^−1^)	9.17 ± 1.23	9.85 ± 0.82	9.48 ± 1.44	9.35 ± 0.98	n.s	n.s	≤ 0.05
V_IAT_ (km h^−1^)	12.0 ± 1.19	13.7 ± 0.87***	12.2 ± 1.46	12.8 ± 0.85*	≤ 0.001	n.s	≤ 0.01
Lactate _LT_ (mmol l^−1^)	1.85 ± 0.78	1.23 ± 0.40**	2.33 ± 1.38	1.54 ± 0.68**	≤ 0.001	n.s	n.s
Lactate _IAT_ (mmol l^−1^)	3.37 ± 0.79	2.73 ± 0.40**	3.84 ± 1.38	3.04 ± 0.68**	≤ 0.001	n.s	n.s
Body weight (kg)	78.5 ± 9.2	77.0 ± 9.0*	75.6 ± 7.7	74.9 ± 7.5	≤ 0.01	n.s	n.s
FM (kg)	15.0 ± 4.0	13.3 ± 4.3**	13.5 ± 4.6	12.3 ± 4.6*	≤ 0.001	n.s	n.s
FFM (kg)	63.5 ± 7.3	63.7 ± 6.9	62.1 ± 4.8	62.6 ± 4.6	n.s	n.s	n.s
FM (%)	18.9 ± 4.2	17.2 ± 4.4**	17.5 ± 4.8	16.1 ± 5.0*	≤ 0.001	n.s	n.s
FFM (%)	81.1 ± 4.2	82.8 ± 4.5	82.5 ± 4.9	83.9 ± 5.1	≤ 0.001	n.s	n.s

*TG* Treatment group, *CG* Control group, *V*_*LT*_ Velocity at lactate threshold, *V*_*IAT*_ Velocity at individual anaerobic threshold, *LT* Lactate threshold, *IAT* Individual anaerobic threshold, *HR* Heart rate, *FM* Fat mass, *FFM* Fat free mass, *T* Main time effect, *G* Main group effect, *T* × *G* Time and group interaction effect

*p ≤ 0.05; **p ≤ 0.01. ***p ≤ 0.001 within the group from baseline to final examination

#### Time Trial

**Fig. 2:**
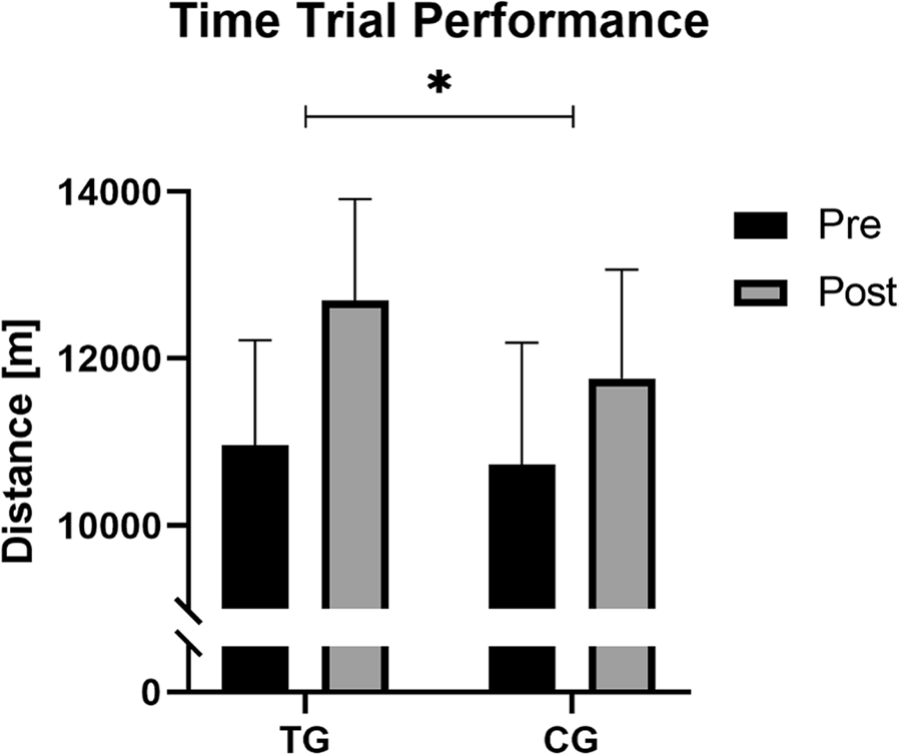
Running distance in the 60min time trial before (pre) and after (post) 12 weeks of intervention in TG (*n* = 15) and CG (*n* = 17). *Significantly different (*p* ≤ 0.05) by mixed ANOVA (time × group interaction)

The current investigation identified a statistically significant improvement in time trial performance in both intervention groups (Table 4). The results of the mixed ANOVA showed that the TG exhibited a statistically significant (*F*_1,30_ = 5.409, *p* ≤ 0.05; *η*_p_^2^ = 0.153) greater increase in running distance (1727 ± 705 m) compared to the CG (1018 ± 976 m), as shown in Fig. 2. The additional increase in running distance by SCP supplementation was also reflected by the large effect size. Analysis with unpaired *t*-tests revealed that the two groups did not differ significantly in heart rate. Furthermore, their ratings of perceived exertion at any measurement time point during and after the 60-min time trial showed no significant group differences.

#### Incremental Running Test and Blood Lactate Analysis

**Fig. 3:**
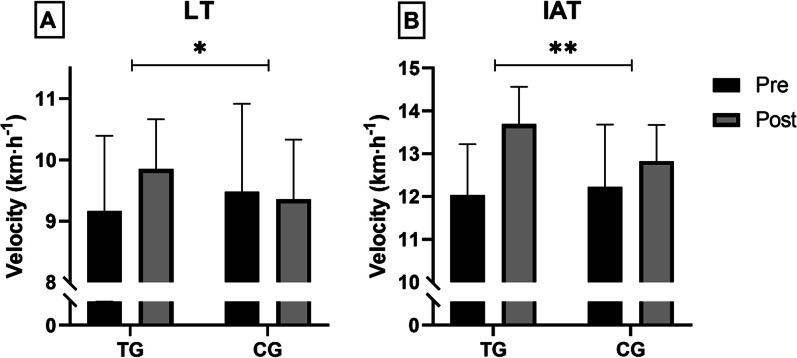
Velocity at LT (**A**) and at IAT (**B**) before (pre) and after (post) 12 weeks of intervention in TG (*n* = 15) and CG (*n* = 17). *Significantly different (*p* ≤ 0.05) **Significantly different (*p* ≤ 0.01) by mixed ANOVA (time × group interaction)

After 12 weeks, the velocity at LT increased in the TG (0.68 ± 1.27 km h^−1^_;_ n.s.) and decreased slightly in the CG (− 0.14 ± 0.98 km h^−1^; n.s.), resulting in a statistically significant difference between these two groups (*F*_1,30_ = 4.067, *p* ≤ 0.05; *η*_p_^2^ = 0.119) as demonstrated in Fig. 3.

The velocity at IAT improved in the TG (1.660 ± 1.022 km h^−1^; *p* ≤ 0.001), but also in the CG (0.606 ± 0.974 km h^−1^; *p* ≤ 0.05). Differences between groups were statistically significant and meaningful as described by the large effect size (*F*_1,30_ = 8.912, *p* ≤ 0.01; *η*_p_^2^ = 0.229, Fig. 3). Analysis with unpaired *t*-tests revealed that the two groups did not differ significantly in heart rate. Furthermore, their ratings of perceived exertion at any measurement time point during and after the 60-min time trial showed no significant group differences.

Regarding blood lactate accumulation, a reduction in lactate levels at the LT was observed in both, the TG (− 0.627 ± 0.681 mmol l^−1^; *p* ≤ 0.01) and CG (− 0.796 ± 1.06 mmol l^−1^; *p* ≤ 0.01), with no significant group difference (*F*_1,30_ = 0.277, n.s.; *η*_p_^2^ = 0.009, Table 4).

Comparable results were obtained from the lactate levels at the IAT. In both groups, lactate concentrations decreased (TG: − 0.638 ± 0.689 mmol l^−1^; *p* ≤ 0.01 and CT: − 0.795 ± 1.06 mmol l^−1^; *p* ≤ 0.01) without significant group difference (*F*_1,30_ = 0.240, n.s.; *η*_p_^2^ = 0.008, Table 4).

#### Body Composition

Following the intervention, body weight decreased by − 1.5 ± 2.2 kg in TG on a statistically significant level (*p* ≤ 0.05) and by − 0.7 ± 1.9 kg in CG (n.s.). However, there were no statistically significant group differences (*F*_1,30_ = 1.348, n.s.; *η*_p_^2^ = 0.043) as shown in Table 4.

In the TG, FM was reduced by − 1.7 ± 1.6 kg (*p* ≤ 0.01) and in the CG by − 1.2 ± 2.0 kg (*p* ≤ 0.05). The different outcome between the two treatment groups was not statistically significant as evidenced by ANOVA (*F*_1,30_ = 0.601, n.s.; *η*_p_^2^ = 0.020).

Translating these findings in relative percentage FM values, a significant decline of FM was observed in both TG (− 1.7 ± 1.7%; *p* ≤ 0.01) and CG (− 1.4 ± 2.3%; *p* ≤ 0.05). However, no significant group differences (*F*_1,30_ = 0.130, n.s.; *η*_p_^2^ = 0.004) were identified.

On average, participants gained 0.2 ± 1.2 kg (n.s.) and 0.5 ± 1.3 kg (n.s.) FFM in the TG and CG, respectively. Furthermore, mixed ANOVA revealed no significant time (*F*_1,30_ = 2.287, n.s.; *η*_p_^2^ = 0.071), group (*F*_1,30_ = 0.374, n.s.; *η*_p_^2^ = 0.012), or interaction (*F*_1,30_ = 0.409, n.s.; *η*_p_^2^ = 0.013) effects in absolute FFM (Table 4).

The TG increased relative FM by 1.7 ± 1.7% (*p* ≤ 0.01) and the CG by 1.4 ± 2.3% (*p* ≤ 0.05). The different outcome between the two treatment groups was not statistically significant as evidenced by ANOVA (*F*_1,30_ = 0.447, n.s.; *η*_p_^2^ = 0.015).

### Physical Activity and Dietary Intake

As summarized in Table 5, the groups did not differ significantly in changes in energy expenditure, total energy intake, and intake of the macronutrients, protein, fat, and carbohydrates during the intervention.

**Table 5:** Changes in daily energy expenditure and dietary intake of total energy and macronutrients before (PRE) and following (POST) the intervention with supplementation of SCP (TG) or placebo (CG)

Variables	TG (*n* = 15)		CG (*n* = 17)		*p* (mixed ANOVA)		
	PRE	POST	PRE	POST	*T*	*G*	*T* × *G*
Energy expenditure (kca/day)	4506 ± 2569	4063 ± 3686	5138 ± 2668	3910 ± 2685*	n.s	n.s	n.s
Total energy intake (kcal/day)	2103 ± 746	1914 ± 913	2676 ± 646	2801 ± 872	n.s	≤ 0.01	n.s
Protein intake (g/day)	84.8 ± 32.9	78.7 ± 38.0	113 ± 41.6	97.5 ± 31.3	≤ 0.05	n.s	n.s
Fat intake (g/day)	97.5 ± 77.3	90.3 ± 87.0	104 ± 21.9	99.6 ± 32.1	n.s	n.s	n.s
Carbohydrate intake (g/day)	241 ± 127	239 ± 108	283 ± 78.7	296 ± 124	n.s	n.s	n.s

*TG* Treatment group, *CG* Control group, *FM* Fat mass, *FFM* Fat free mass, *T* Main time effect, *G* Main group effect, *T* × *G* Time and group interaction effect

*p ≤ 0.05; within the group from baseline to final examination

Only the CG showed significantly higher total energy intake over both measurements compared to the TG.

## Discussion

The main finding of the present study was that supplementation with SCP in combination with CT was associated with a significant increase in time trial performance compared to CT alone. Furthermore, a significantly higher velocity at the LT and IAT during an incremental treadmill test was observed. Moreover, the SCP supplementation combined with CT seemed to have a slightly greater non-significant impact on reductions in absolute and relative body fat mass than CT alone.

Although the close sequential combination of different training modalities within CT promises efficient improvements in strength and endurance performance, a compromising effect of the combination on adaptations compared to training both exercise modalities alone is commonly discussed [10, 44, 45]. This so-called interference effect seems mostly to be mediated by the inhibition of molecular signaling pathways from each type of training [10, 11], and its magnitude seems to be greatly influenced by individual training variables [10, 45]. However, the interference typically becomes evident as compromised strength, hypertrophy or power development adaptions compared with resistance training alone [46–49]. Resistance training within CT, on the other hand, appears to have minimal to no negative effects on endurance performance or aerobic capacity [8, 50]. The training modalities used in the present study show a focus on continuous endurance training. Thus, interference effect does not appear to represent a substantial influence on endurance performance adaptations as the main outcome parameter.

The extent and manner in which prolonged protein intake can improve performance, and endurance performance in particular has long been debated. The effects of SCP on endurance performance in the present trial are supported by a recent meta-analysis by Lin and colleagues [51]. This quantitative approach examined both endurance training-only studies and CT programs. Including a total of n = 19 studies, the authors found that protein supplementation improved time trial performance compared to controls that performed endurance training or CT only. Furthermore, greater improvements in VO_2_peak and peak workload power were observed [51].

In a previous study, a 12-week CT combined with the supplementation of 15 g of SCP showed a positive effect on time trial performance in women [20].

However, a recent meta-analysis has concluded that protein supplementation did not increase aerobic capacity or VO2max/VO2peak following CT in the studies included [7]. With regard to possible mechanisms responsible for the improvements in endurance performance observed in this study, different models of adaptation could be discussed. Although it was a CT regimen, endurance training was a major component of the training (60 min/session in the range of the anaerobic threshold).

The increase in time trial performance by 15% goes in parallel with the percentage increase in the speed at the individual anaerobic threshold (+ 14%), indicating that this was most likely crucial for the improved time trial performance. Lower lactate concentrations can be the result of decreased production or increased clearance. Lactate levels were lower at the LT and IAT in both groups as a result of the training program; however, there were no significant time*group interaction effects indicating a superior effect of SCP supplementation on the overall lactate metabolization. In contrast, the velocity at the aerobic threshold as well as the speed at the anaerobic threshold were significantly higher in the SCP group. In general, a rightward shift in lactate thresholds is attributed, at least in part, to a training-induced change in fuel utilization toward aerobic carbohydrate and fat metabolism, which is associated with, among other things, increased mitochondrial content and thus, improved aerobic capacity [52–56]. In a preclinical controlled trial with a rat model, a significantly increased mitochondrial density has been determined by transmission electron microscopy after 4 weeks of oral SCP supplementation. This effect was not detectable in rats fed with the tap water control, which might be indicative for improved aerobic metabolism by the supplementation of SCP [57].

Considering the fact that the exact metabolic or structural adaptations in aerobic metabolism by SCP have not been elucidated, it is possible that an improved running economy could be responsible for the present findings. An augmentation in endurance running performance may not exclusively be attributed to aerobic and anaerobic capacity but also to neuromuscular motor competence and running economy [58, 59].

In a recent meta-analysis by Trowell et al. [60], the authors reported that CT programs are effective in improving force-generating capacity of the ankle plantar flexors. Given that human tendons are composed of 60–85% collagen [61], collagen peptide supplementation could be a promising strategy to improve musculotendinous properties. Structural changes resulting, e.g., in an altered musculotendinous stiffness might contribute to an improved running economy which might be explained by an augmented energy storage capacity of the myotendinous system [60]. Praet et al. [62] have shown that SCP administration significantly improves Achilles tendon function in patients with tendinopathy. Jerger et al. [63] have demonstrated that supplementation with SCP and resistance exercise was associated with a greater hypertrophy in tendinous and muscular structures than resistance training alone. In a most recent muscle biopsy study, SCP induced a significantly higher upregulation of key anabolic pathways in human skeletal muscle 4 h following an acute resistance training compared to a placebo control group that underwent the same training regime. This effect could be responsible for chronic beneficial adaptations of musculotendinous structures [21].

Therefore, it could be speculated that the differences in running performance might partly be explained by positive changes in structural and mechanical properties of the muscle–tendon system following CP supplementation leading to an improved running economy [62]. In the current investigation, the TG showed slightly larger increases in fat free mass compared to CG (1.7% vs. 1.4%). But in contrast to previous studies [13, 15, 17], there were no statistically significant differences between the groups. It has to be mentioned that muscle hypertrophy was not the aim of the current investigation.

In general, differences in total energy or macronutrient provision throughout the study, including the level of glycogen stores, could also be a relevant factor influencing time trial or ergometric performance. However, the nutritional protocols have not shown any significant difference in energy or macronutrient uptake and the standardized breakfast protocol has minimized differences in the availability of macronutrients during exercise.

The improvements in indicators of endurance capacity in the present study by CT and SCP supplementation may represent a promising approach to improve endurance capacity in athletes. Moreover, an effect on aerobic metabolic processes during exercise could potentially lead to changes in the body composition of exercisers in the long term, such as a reduction in fat mass. Nevertheless, since the study design does not provide direct insights into underlying mechanisms and these are not known so far, further studies are needed for a deeper understanding and functional conclusions.

However, it must also be noted that the present investigation has some limitations. First, due to the high dropout rate, the size of the analyzed population is rather small. Secondly, wind conditions were not recorded in the present trial. However, potential biases in weather conditions were accounted for by implementing randomized testing days for TG and CG. In addition, in the present trial shows a discrepancy between caloric intake and energy expenditure. This might be partly explained by underreporting of dietary intake [64] and overreporting of physical activity [65]. Furthermore, although SCP supplementation in combination with CT is more effective in improving running performance and indicators of endurance capacity in recreational men than CT alone, the present results should be viewed with caution when extrapolated to clinical or athlete populations, as training-induced adaptive responses to CT and protein supplementation are largely driven by training status [12]. Therefore, further studies with endurance athletes are needed to investigate the potential underlying physiological and molecular mechanisms, e.g., using muscle biopsies. In addition, it has also to be pointed out that the results presented are only valid for the specific collagen peptide composition used in this study. Other collagen derived products might exhibit disparate pharmacological effects due to differences in composition [66].

## Conclusion

In summary, the results of this placebo-controlled trial showed that SCP supplementation in combination with CT improved indicators of endurance capacity in response to a 12-week concurrent training program in recreationally active men. The main result was a significant improvement in a 1-h time trial by 15%. In addition, the velocity at the aerobic and individual anaerobic threshold increased significantly in the participants receiving 15 g SCP on a daily basis. Therefore, daily supplementation with SCP could positively enhance the adaptions of a CT program, which are often lower compared to isolated endurance or strength training due to the interference effect. The results could potentially be explained by an improved aerobic capacity or structural adaptations within the musculotendinous structures, or a combination of both effects. However, this needs to be confirmed and further investigated by forthcoming studies.

## Data Availability

The datasets generated and/or analyzed during the current study are not publicly available but are available from the corresponding author on reasonable request.

## Abbreviations

BIA: Bioelectric impedance analysis
BMI: Body mass index
CG: Control group
CT: Concurrent training
ECM: Extracellular matrix
FAM: Familiarization
FFM: Fat free mass
FM: Fat mass
HR: Heart rate
IAT: Individual anabolic threshold
LT: Lactate threshold
M: Mean
PLA: Placebo
ANOVA: Analysis of variance
RPE: Rating of perceived exertion
SCP: Specific collagen peptides
SD: Standard deviation
TG: Treatment group
*V*_IAT_: Velocity at individual anabolic threshold
*V*_LT_: Velocity at lactate threshold
WHO: World Health Organization
